# Incompletely observed: niche estimation for six frequent European horsefly species (Diptera, Tabanoidea, Tabanidae)

**DOI:** 10.1186/s13071-020-04316-7

**Published:** 2020-09-10

**Authors:** Dorian D. Dörge, Sarah Cunze, Sven Klimpel

**Affiliations:** 1 Institute for Ecology, Evolution and Diversity, Goethe-University, Max-von-Laue-Str. 13, 60439 Frankfurt/Main, Germany; 2 Senckenberg Biodiversity and Climate Research Centre, Senckenberg Gesellschaft für Naturforschung, Senckenberganlage 25, 60325 Frankfurt/Main, Germany

**Keywords:** Tabanidae, *Tabanus*, *Haematopota*, *Chrysops*, Niche, Climate, Land cover, Surface range, Model, Envelope

## Abstract

**Background:**

More than 170 species of tabanids are known in Europe, with many occurring only in limited areas or having become very rare in the last decades. They continue to spread various diseases in animals and are responsible for livestock losses in developing countries. The current monitoring and recording of horseflies is mainly conducted throughout central Europe, with varying degrees of frequency depending on the country. To the detriment of tabanid research, little cooperation exists between western European and Eurasian countries.

**Methods:**

For these reasons, we have compiled available sources in order to generate as complete a dataset as possible of six horsefly species common in Europe. We chose *Haematopota pluvialis*, *Chrysops relictus*, *C. caecutiens*, *Tabanus bromius*, *T. bovinus* and *T. sudeticus* as ubiquitous and abundant species within Europe. The aim of this study is to estimate the distribution, land cover usage and niches of these species. We used a surface-range envelope (SRE) model in accordance with our hypothesis of an underestimated distribution based on Eurocentric monitoring regimes.

**Results:**

Our results show that all six species have a wide range in Eurasia, have a broad climatic niche and can therefore be considered as widespread generalists. Areas with modelled habitat suitability cover the observed distribution and go far beyond these. This supports our assumption that the current state of tabanid monitoring and the recorded distribution significantly underestimates the actual distribution. Our results show that the species can withstand extreme weather and climatic conditions and can be found in areas with only a few frost-free months per year. Additionally, our results reveal that species prefer certain land-cover environments and avoid other land-cover types.

**Conclusions:**

The SRE model is an effective tool to calculate the distribution of species that are well monitored in some areas but poorly in others. Our results support the hypothesis that the available distribution data underestimate the actual distribution of the surveyed species.
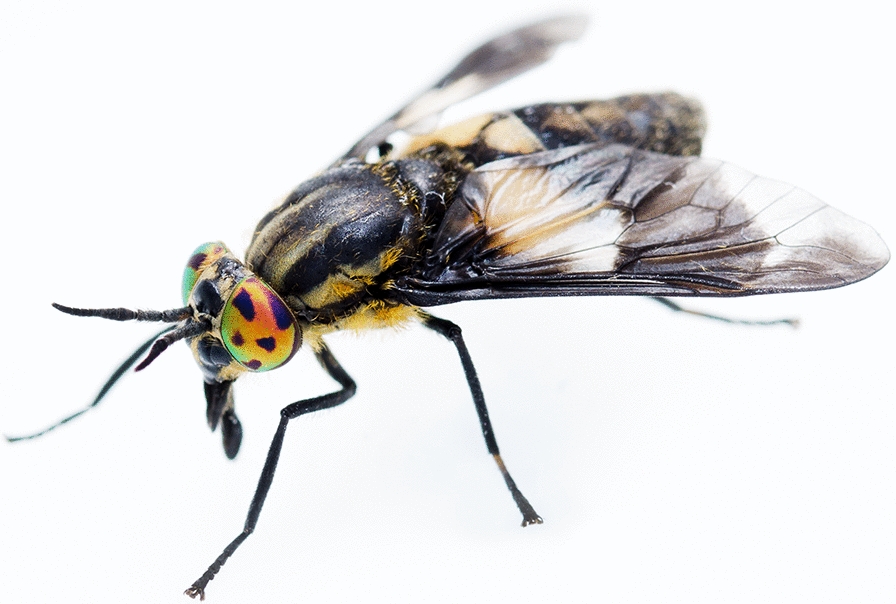

## Background

Common throughout the world, tabanids are hematophagous dipterans. Worldwide there are about 4400 known species [1, 2] of which more than 170 occur in Europe [3]. Female horseflies can cause severe skin lesions [4, 5] and are able to effectively transmit different diseases [6–8] due to their excessive feeding behavior [9]. These include the eye worm *Loa loa* (sausing loaiasis) [2, 7, 10, 11], the equine infectious anemia virus [12–14], *Trypanosoma theileri* [15, 16] and *T. evansi* (Surra) which mainly infect livestock [2] but can also infect humans [17]. Further transmittable pathogens are *Spiroplasma* [18–20], *Bacillus tularensis* (causing tularemia) [21], *Bacillus anthrax* (Anthrax) [12], bovine mycoplasma [22], *Elaeophora schneideri* (causing elk and deer filariosis) [23] as well as *Besnoitia besnoiti* (causing bovine besnoitiosis) [24].

Many species require slow flowing or stagnant water with shallow zones for egg-laying and for the migration of larvae between land and water. The larvae live predatorily or feed on detritus at the edge of the water, seeking dry ground to pupate. Other species, however, are specialized in drier areas and do not require bodies of water but only moist soil or dung from grazing animals [2, 3, 25–27]. As a result of the draining of many of Europe’s wetlands [28, 29], the number of susceptive horseflies has fallen sharply [30]. Current insecticide- and land-use changes are further reducing the numbers [31–34]. However, especially in poorer countries, cattle and other livestock continue to suffer due to lack of protection or control options, resulting in anemia or severe skin damage to the affected animals [2, 35, 36].

Recent research within Europe is focused mainly on monitoring points within a few countries for the occurrence of horseflies and potential control measures [37] as well as ecological and anthropogenic effects on their populations [38]. To date, there are no standardized and repeatedly executed monitoring protocols for horseflies in Eurasia (and other continents as well), which makes it difficult to acquire, compile and utilize existing data for calculations and projections. Due to the different monitoring schemes within different countries, occurrences are either over- or underestimated and combining these datasets is complicated. Based on the lack of monitoring in many countries, not much is known about horsefly complete distribution. Finally, since only sites in western Europe have been extensively recorded, the distribution in the rest of Eurasia is most likely greatly underestimated.

Six species commonly observed in central Europe were used for our study: *Chrysops relictus* (Meigen 1820) and the morphologically similar species *Chrysops caecutiens* (Linnaeus, 1758), *Haematopota pluvialis* (Linnaeus, 1758), *Tabanus bromius* (Linnaeus, 1758), *Tabanus bovinus* (Linnaeus, 1758) and *Tabanus sudeticus* (Zeller, 1842). *Tabanus* spp. and *Haematopota* spp. are relatively eurytopic and do not require stagnant water but moist soil for egg-laying and larval development [25, 39–43], while *Chrysops* belongs to the hydrophilous ecological group and depends on ponds, rivers or lakes [44].

To find a realistic dispersal of the species, we calculated the climatic niche and the land cover allocation of occurrence points using available literature and database data ranging back to 1990. We used the ecological niche model (ENM) with a surface-range envelope (SRE) to project the potential distribution within Europe and Asia. In order to counteract the present sampling bias, we used this method, as it is particularly resistant to over- and under-representation of species in databases and literature. We also compared the modelled niches (climatic envelopes), as well as the preferred type of land cover and the number of frost-free months required for the six species to exist.

## Methods

For our analysis, we compiled data collected from an extensive literature research [37, 45–113] as well as the GBIF-Database [114–120]. Occurrence data were adjusted to the spatial resolution (5 arc-minutes) of the environmental raster data and reduced to one occurrence per grid cell.

### Estimation of the potential distribution

For the niche range analysis, 8 bioclimatic variables provided by Worldclim [121] were downloaded at a spatial resolution of 5 arc-minutes. The variables Bio5, Bio6, Bio13, Bio14, Bio18 and Bio19 were used. We computed SREs (as implemented in the *biomod2* R-package [122] for each tabanid species and considered three models: the full model (yellow in the depictions), 95% (orange) and 90% (red) of all occurrence points. Maps were created in Esri ArcGIS [123].

### Comparison of requirements

Data were acquired from ESA GlobCover [124] for the activity phases, as well as for the land-cover preference comparisons. For the activity comparison, the amount of frost-free months was derived from the monthly minimum temperature, provided by Worldclim [121]. The type of land cover was obtained from GlobCover at the respective sites for the land-cover comparison and the relative frequencies of individual LC-types were compared with the availability of the LC-type (relative frequency in the study area). The range of the study area is reduced to −10°W, 45°E, 79°N and 35°S based on the lack of data from more eastern areas. Land cover categories were combined when adequate, resulting in 11 categories: Cropland > 50% (11, 14); Grass/Shrubland (110, 120, 130, 140); Broadleaf Forest (40, 50, 60); Mixed Forest (100); Dense Evergreens (70); Light Evergreens (90); Mosaic Vegetation (20, 30); Sparse Vegetation (150); Artificial (190); Water Bodies (210); and Other (160, 170, 180, 195, 215).

## Results

**Fig. 1:**
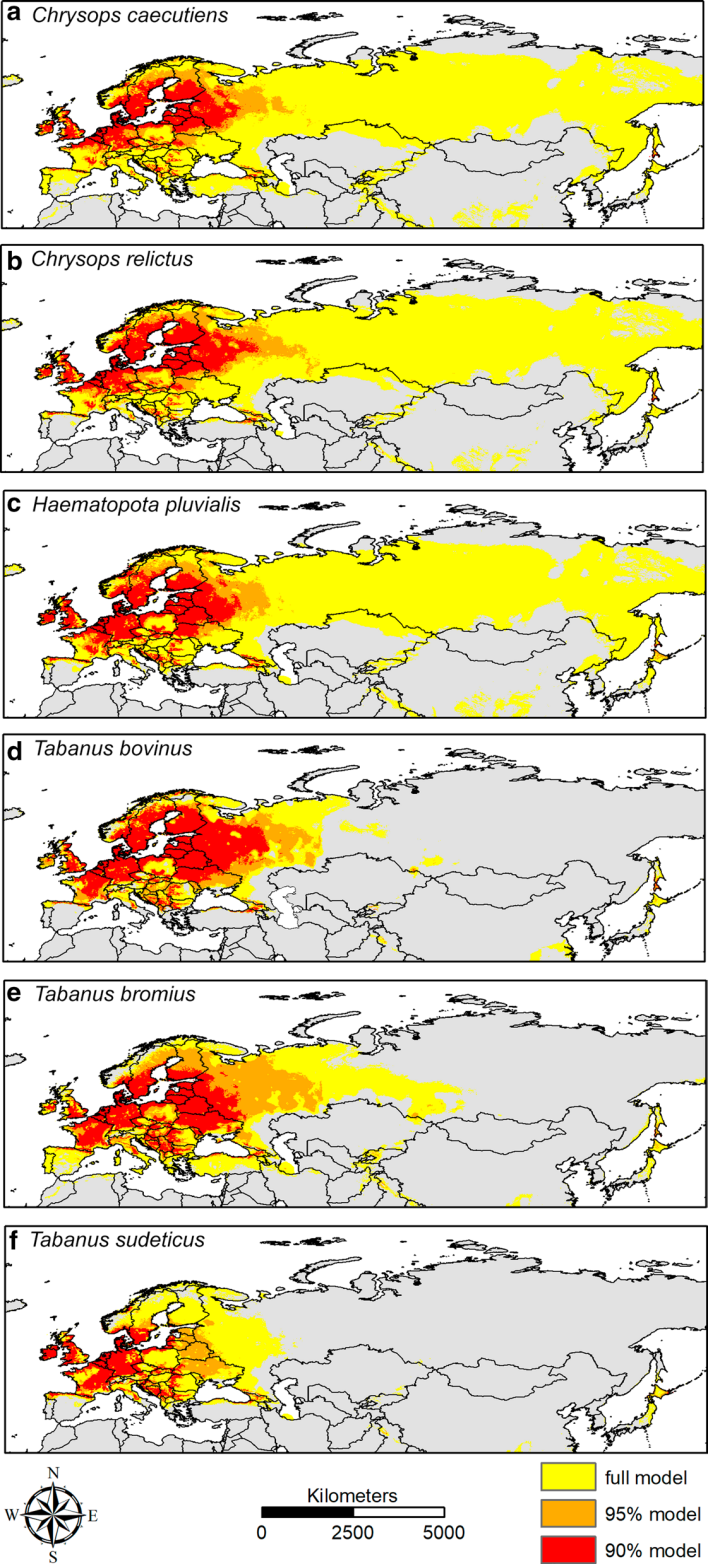
Modelled distribution of the six species. *Key*: yellow, full model; orange, 95% model (5% outliers removed); red, 90% model (10% outliers removed). Figure created with Esri ArcGIS [123]

**Fig. 2:**
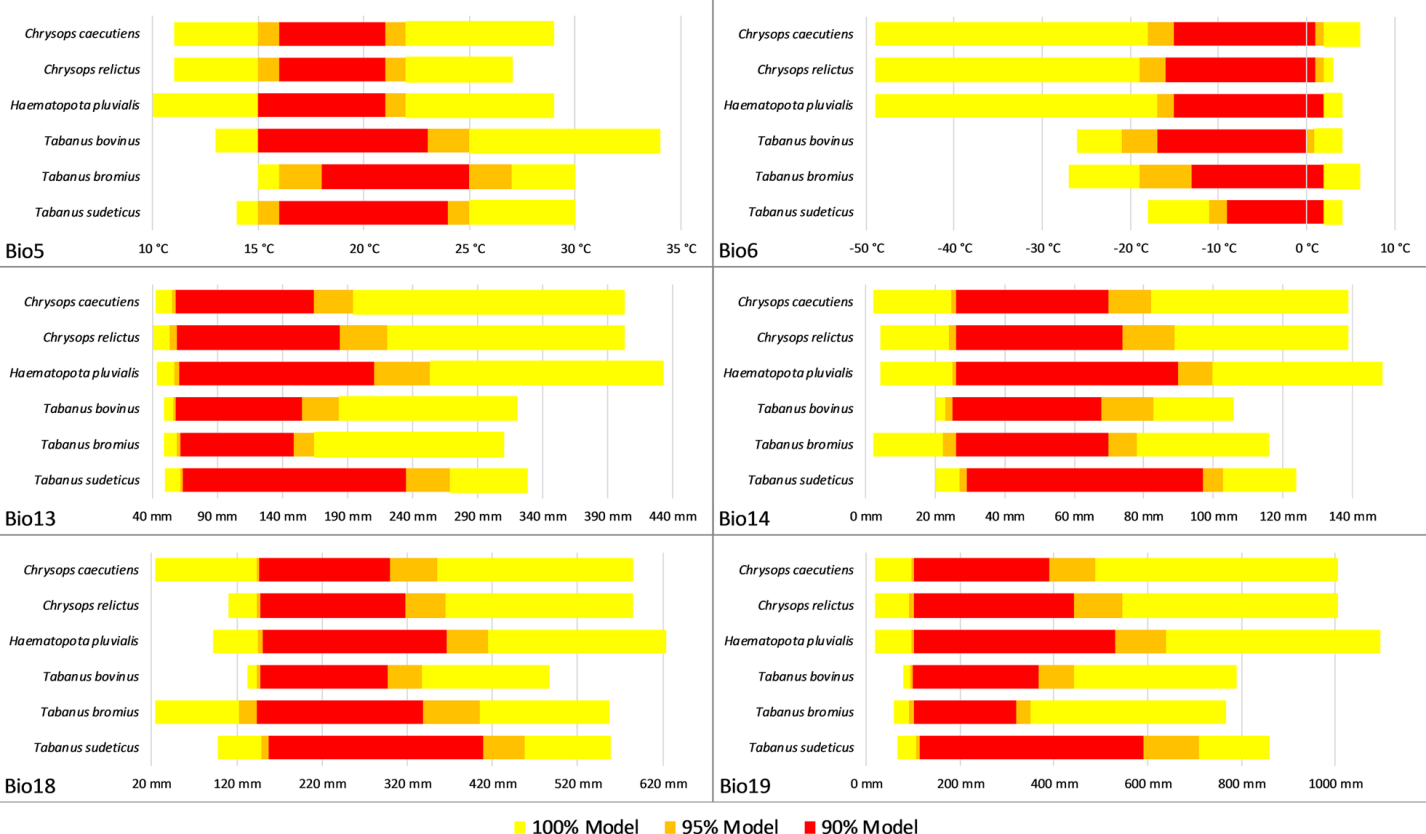
Comparison of the modelled niches for the six species, *Chrysops caecutiens*, *C. relictus*, *H. pluvialis*, *T. bovinus*, *T. bromius* and *T. sudeticus*, in different climatic variables. *Abbreviations*: Bio5, maximum temperature of warmest month; Bio6, minimum temperature of coldest month; Bio13, precipitation of wettest month; Bio14, precipitation of driest month: Bio18, precipitation of warmest quarter; Bio19, precipitation of coldest quarter. *Key*: yellow, full model; orange, 95% quantile model; red, 90% quantile model

Figure 1 shows three different models of all six surveyed species. The 90% and 95% models for *C. caecutiens* showed a very fragmented distribution with the center of these models lying in the northern part of Europe. The full model extended from central Spain over all European countries, including Turkey and Russia, as far as the eastern part of Siberia. A very similar picture emerged for *C. relictus* and *H. pluvialis*, where only the areas in Spain and Turkey are missing in the comparison. Incorporating the niches’ climatic variables (Fig. 2), all three species showed very similar patterns: the 90% and 95% model mostly made up less than 50% of the full model and were skewed in one direction. In climatic variable Bio18, *C. caecutiens* showed a higher tolerance for low precipitation than *C. relictus* and *H. pluvialis*.

For *T. bovinus*, *T. bromius* and *T. sudeticus*, the 90% and 95% models were closer to the full model. The full model closed gaps in central Europe as well as added areas in (northeastern) Finland and central Russia. For *T. sudeticus*, the full model closed most gaps within the original distribution. The climatic variables (Fig. 2) were relatively similar for these three species. For *T. sudeticus*, the 95% model incorporated most of the niche when considering only the variables.

**Fig. 3:**
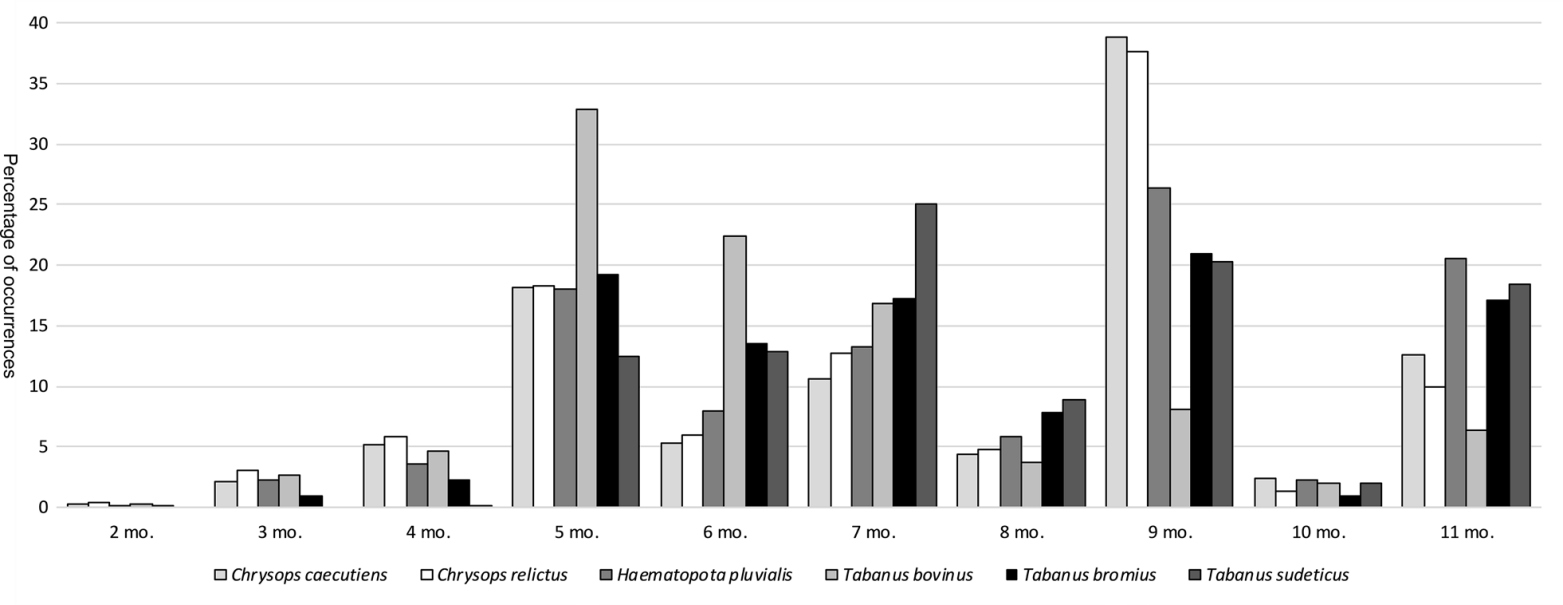
Percentage occurrence as a function of the number of frost-free months. For each species, the sum of all categories equals 100%. *Abbreviation*: mo, months

Figure 3 shows that most species (except *T. sudeticus*) occur in small numbers in areas with two frost-free months. Most occurrences are within 9 months for *C. relictus*, *C. caecutiens* and *H. pluvialis*. *Haematopota pluvialis* also had a slightly decreased occurrence rate of 11 months. The highest numbers of individuals of *T. bovinus* occured at 5 and 6 months. *Tabanus bromius* showed a steady distribution at 5, 6, 7, 9 and 11 months. *Tabanus sudeticus* showed the most individual occurrences at 7 and 11 months. The data from 5 months on (except for 10 months) showed a slightly lower frequency. No species demonstrated more than 3% of their occurrences in areas with 10 frost-free months.

**Fig. 4:**
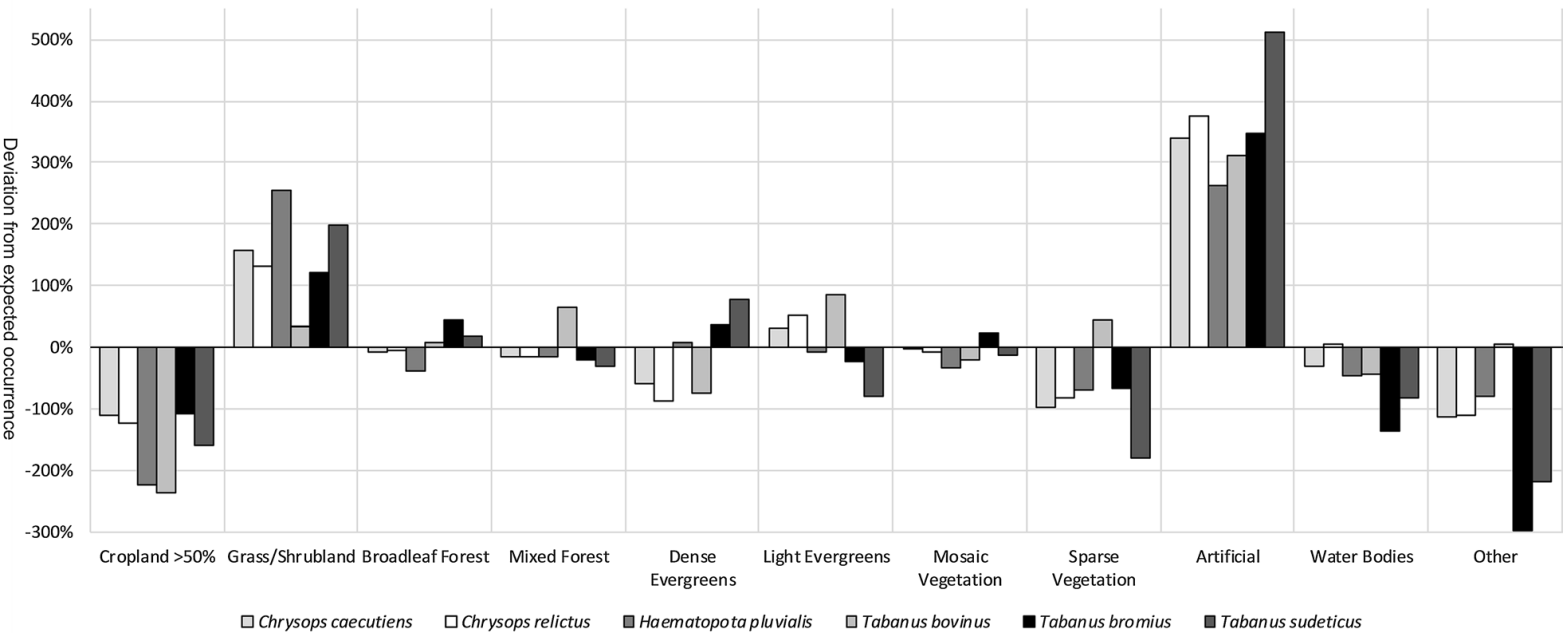
Deviation of occurrence of the species compared to available land cover. A positive value of 100% shows that the species occurs twice as often as expected in the respective areas. Conversely, a negative value of 100% indicates an abundance that is only half as high as expected

The comparison of land cover type and species occurrence (Fig. 4) shows that in the Cropland category, all the species occured at a frequency between half and a quarter of the expected value. Tabanids occured in areas with the category Grass/Shrubland between 2–3.5 times the expected frequency, except for *T. bovinus*, which occured only slightly more frequently. In Broadleaf Forest, there were only minor deviations from the expected value, with *H. pluvialis* occurring slightly less frequently and *T. bromius* occurring slightly more frequently. Similarly, in Mixed Forest there was only a slightly higher value for *T. bovinus*. In the category Dense Evergreens, *C. caecutiens*, *C. relictus* and *T. bovinus* showed a negative deviation from the expected value between 60% and 90% while *T. bromius* (30%) and *T. sudeticus* (80%) were more common. Except for the values, this effect was exactly the opposite in the category Light Evergreens. Mosaic vegetation shows no fundamental difference. Sparse Vegetation showed a slight increase in occurrence of *T. bovinus*, but a reduction of the other species between 60–180%. The Artificial category showed the largest deviations from the expected value by far, with positive deviations between 260% (2.6 times the expected value) and 510% (5.1 times the expected value). In the Water Bodies category, the values were slightly negative for *C. caecutiens*, *C. relictus* and *T. bovinus*, while they are more pronounced for the species *T. bromius* (130%) and *T. sudeticus* (80%). The category Other showed medium to strong negative deviations for all species except for *T. bovinus*.

## Discussion

We modelled the potential distribution of six common horsefly species in Eurasia and compared their niches. An SRE model was used because no extensive monitoring with standardized methods exists. Hence, the available data show a strong bias with large regions being severely underrepresented or not considered at all. Due to the very dense sampling in western Europe, a skewed picture emerges, although several of the species also occur about 6000 km further east. The investigated species require moist soil (*Tabanus*, *Haematopota*) or lakes, ponds and rivers (*Chrysops*) for egg deposition and larval development [25, 39–42]. In addition, the larvae are often detrivorous or can feed predatorily on small insects or worms [125]. The species are relatively common and widespread in Europe and are therefore likely to appear in many surveys, making them adequate examples for this methodology. For the model, we counteracted the sampling bias as much as possible by reducing the number of samples to one per grid cell. It is therefore likely that all species can truly fill most of the niche (full model) calculated in the analysis.

When comparing the areas of the 90% model, it becomes apparent that the distribution area is very small due to a dense monitoring in western and central Europe and a very similar distribution for all six species could be expected. When taking the full model into account, a different picture emerges. Three species, i.e. *C. caecutiens*, *C. relictus* and *H. pluvialis*, have a much larger niche than evident from the data. Here, *C. caecutiens* has the largest distribution and the distribution areas of the other three species overlap even in the eastern areas, where only few surveys have been made. *Tabanus bovinus* and *T. bromius* have similarly large niches which are mostly overlapping and are supported by data collection in Europe. *Tabanus sudeticus* has the smallest distribution. The distribution of collected sightings of *T. bovinus* and the results of our calculation are very close to the known distribution which is shown in Fig. 1.

### Activity phases

When comparing the frequency of occurrence as a function of the number of frost-free months, it is apparent that five of the six species can occur in areas with only two frost-free months, albeit with only a few individuals. This frequency gradually increases up to five months, with *T. sudeticus* appearing in areas with at least four frost-free months. The remaining numbers show the direct influence of the sampling bias towards central and western Europe. The extreme peak at nine months is mainly due to heavy sampling in central Europe, while the increased numbers at 11 months are almost entirely due to the inclusion of England and Ireland. It is known that horseflies hibernate as larvae and may require several years for their development [126]. In central Europe, development spans between one and three years. However, assuming an area with only two frost-free months per year, this number could increase significantly. The most cold-tolerant species are *C. caecutiens*, *C. relictus* and *H. pluvialis* with occurrences in areas that plunge below −58 °C.

### Land-cover comparison

As expected, monoculture cropland was avoided by all six species. This may be due to pesticide use, lack of hosts and lack of areas for egg-laying and larval development and lack of adequate sites for mating behavior, as well as a shortage of sugar sources [127–129]. It is also not surprising that grassland and scrubland are preferred. Since Grasslands, or areas with some lowland scrub, are mostly used as grazing land for livestock [130], tabanids can easily find the hosts they need. Broadleaf forest, mixed forest and mosaic vegetation show no particular effect on tabanid preference or aversion. However, Dense Evergreen and Light Evergreen showed an interesting pattern on preference and aversion, which largely balances out when the two categories are combined. We remark that *C. relictus*, *C. caecutiens* and *T. bovinus* avoid dense evergreen, while at least *T. sudeticus* prefers it. Sparse vegetation is avoided by all species except for *T. bovinus*. This can be explained by the fact that within these areas, significantly fewer animals can serve as hosts. An interesting result is that all species have an extreme preference for Artificial areas category. This is most likely due to the fact that populated areas harbor domestic animals, grazing animals, livestock and, ultimately, people in the immediate vicinity. It is important to note that although the dataset has been adjusted and reduced to one point per grid cell, a sampling bias is still present towards heavily populated as well as frequently surveyed areas. This would explain at least part of the extreme values of the Artificial category. Baldacchino et al. [38] were able to show parts of the current horsefly diversity of western and southern European countries in a large-scale study of almost 80,000 captured animals. In comparison to other areas, a significantly lower diversity of species could be found on pastureland, with larger, well-flying species preferring these areas for host searching. Another study by Baldacchino et al. [113] also suggested a preference for mosaic landscape and light forest. Our analysis cannot confirm this result since our dataset does not support any preference for mosaic landscape. On the other hand, our analyses show that forest cover presents mixed results for aversion or preference by the examined species. The land-cover analysis also shows that tabanids equally colonize water bodies if they are available. However, the numbers mostly show an underrepresentation, which is explained by the fact that the available land cover is taken with a resolution of 300 meters, so most water bodies are not presented in the dataset. The category “Other” consists of several land-cover types with very few occurrences and should therefore, be considered carefully if at all. Overall, we have reduced the influence of sampling biases as much as possible, but the effects still shift our results. A standardized monitoring programme is needed to clarify these results and enable future calculations to be more exact.

### Quality of the model

Our envelope model included Japan as a suitable area for all species. This is highly unlikely, at least for the three *Tabanus* species. According to the GBIF database, *H. pluvialis* occurs in Japan. However, this isolated occurrence was not included in the calculation due to the extreme distance to other sites but is a realistic occurrence point for this species after calculating the model. Other remote areas such as the Asian Highlands (Pamir, Hindukush, Himalaya) were additionally estimated as suitable sites by our model. We doubt that these mountain ranges are actually suitable areas for tabanid habitation and that an exclusionary factor is lacking in the model. For the three *Tabanus* species specifically, it is very unlikely that they can be found in these areas. For *Chrysops* species and *H. pluvialis*, however, the areas are within the range of the main distribution spectrum but are discontinuous. We considered temperature and precipitation as important climatic factors. There can also be other factors that are not considered in this study, but which locally exclude the occurrence of these species (e.g. snow cover, humidity). Our model is based on a continental scale, where climatic factors are the most important to show rough distribution patterns [131]. Fine-scale models could go into more detail and include microclimatic effects, but due to the continental scale and the lack of available data, this is beyond the scope of this study. The delimited parts of the model (e.g. southern China, mountain ranges of Asia) in which some species could occur due to a calculated suitable habitat, but either do not occur or it is unknown, show possible distribution areas, which, however, have not been colonized due to dispersal barriers or a missing limiting factor.

## Conclusions

The distribution of most tabanids is not monitored enough in many areas. The SRE model is an effective tool to calculate the distribution of species that are well monitored in some areas but poorly in others. Our results support the hypothesis that the available distribution data underestimate the actual distribution of the surveyed species. Especially *C. relictus*, *C. caecutiens* and *H. pluvialis* have a much larger calculated niche than the collated observations represent. Our results also show that five of the six species occur in areas with only two frost-free months per year, revealing a strong resistance against temperatures up to −58 °C. We found that the six species of horseflies strongly prefer populated areas, as well as grassland and scrubland and avoid arable land and regions of sparse vegetation. Our results reveal that only the observed distribution of *T. bovinus* closely resembles the calculated niche while the other species are most likely not monitored enough. Both *Chrysops* species have almost the same observed distribution and calculated niche, as well as land-cover preferences. We also suggest a standardized monitoring programme, which can improve and validate this methodology for tabanids and other species. With the help of predictions from this model, further monitoring can be planned in areas where few or no observations have been recorded to confirm and extend our model.

## Data Availability

The data are available through the cited references as stated in the Methods section.
